# Soil water availability and evaporative demand affect seasonal growth dynamics and use of stored water in co-occurring saplings and mature conifers under drought

**DOI:** 10.1007/s00468-016-1468-4

**Published:** 2016-09-29

**Authors:** Walter Oberhuber

**Affiliations:** Institute of Botany, Leopold-Franzens-University of Innsbruck, Sternwartestrasse 15, 6020 Innsbruck, Austria

**Keywords:** Conifer, Dendrometer, Mature trees, Radial growth, Sapling, Stem radial variation

## Abstract

**Key message:**

**Differences in temporal dynamics of radial growth and use of stem water reserves in co-occurring saplings and mature conifers are caused by soil water availability and canopy structure**.

**Abstract:**

High-resolution time series of stem radius variations (SRVs) record fluctuations in tree water status and temporal dynamics of radial growth. The focus of this study was to evaluate the influence of tree size (i.e., saplings vs. mature trees) and soil water availability on SRVs. Dendrometers were installed on *Pinus sylvestris* at an open xeric site and on *Picea abies* at a dry-mesic site, and the SRVs of co-occurring saplings and mature trees were analyzed during two consecutive years. The results revealed that irrespective of tree size, radial growth in *P. sylvestris* occurred in April–May, whereas the main growing period of *P. abies* was April–June (saplings) and May–June (mature trees). Linear relationships between growth-detrended SRVs (SSRVs) of mature trees vs. saplings and climate-SSRV relationships revealed greater use of water reserves by mature *P. abies* compared with saplings. This suggests that the strikingly depressed growth of saplings compared with mature *P. abies* was caused by source limitation, i.e., restricted photosynthesis beneath the dense canopy. In contrast, a tree size effect on the annual increment, SSRV, and climate–SSRV relationships was less obvious in *P. sylvestris*, indicating comparable water status in mature trees and saplings under an open canopy. The results of this study provided evidence that water availability and a canopy atmosphere can explain differences in temporal dynamics of radial growth and use of stem water reserves among mature trees and saplings.

**Electronic supplementary material:**

The online version of this article (doi:10.1007/s00468-016-1468-4) contains supplementary material, which is available to authorized users.

## Introduction

The anticipated changes in climate include significant warming in future decades, changes in seasonal precipitation patterns, and an increase in both the frequency and intensity of severe droughts, especially during spring and summer in many mid-latitude regions (IPCC 2013). Hence, we should be aware that drought-exposed forests face an uncertain future, which underline the need to gain more knowledge on tree susceptibility to drought (Allen et al. 2010; Williams et al. 2013; McDowell and Allen 2015). Several authors also reported size-mediated climate sensitivity of forest trees (Mencuccini et al. 2005; Mérian and Lebourgeois 2011; Schuster and Oberhuber 2013a; Bennett et al. 2015), which is expected to affect forest structure, composition, productivity, and climate feedbacks (Bonan 2008). Gaining more knowledge on the influence of tree size on susceptibility to drought will enable us to more accurately model the dynamics of forest ecosystems under climate change.

Drought affects tree water status and impairs radial stem growth, because cell division and cell enlargement require adequate cell turgor (Zweifel et al. 2006; Eilmann et al. 2011; Rossi et al. 2013; Deslauriers et al. 2016). The close coupling of radial stem growth to atmospheric conditions in conifers and broadleaved tree species (Deslauriers et al. 2003; Oberhuber and Gruber 2010; Köcher et al. 2012) points to the importance of tree water status for intra-annual growth of drought-prone conifers (Zweifel et al. 2005; Steppe et al. 2005). Tissue water storage in trees was found to be an important factor in transiently regulating tree water relations (Meinzer et al. 2004; Čermak et al. 2007), and Swidrak et al. (2011, 2014) reported that conifers exposed to drought at the start of the growing season exploit water reserves in the stem to allow temperature-induced growth resumption to occur.

Time series of stem radius variations (SRVs) recorded by automated dendrometers are composed of two components: (i) reversible stem shrinking and swelling caused by dynamics in water storage in elastic tissues outside the cambium, and (ii) irreversible radial stem growth (e.g., Deslauriers et al. 2007; Drew and Downes 2009; Steppe et al. 2015). The reversible component of changes in stem size, i.e., SRV detrended for growth, is the result of changing water potential gradients within the stem and is a measure of water translocation from the elastic tissues outside the cambium to the xylem and vice versa (Zweifel et al. 2001; Sevanto et al. 2011; De Swaef et al. 2015). Tracheid enlargement is regarded as the major driving force of radial stem growth, i.e., the irreversible component in dendrometer traces (Deslauriers et al. 2003). Although water status may strongly affect SRV, especially in slow-growing trees, (Zweifel and Häsler 2001), Linares et al. (2009), and Oberhuber and Gruber (2010) reported a close agreement in intra-annual radial growth determined using dendrometers and xylem cell development. Intra-annual radial growth was also reported to peak synchronously in co-occurring early and late successional conifers (Cuny et al. 2012; Oberhuber et al. 2014). Both components, i.e., reversible tree water status and irreversible radial growth, were found to be controlled by environmental factors on a daily timescale (e.g., Deslauriers et al. 2003; Köcher et al. 2012).

A multitude of studies have employed dendrometers to study water relations and temporal growth dynamics (for a review, see De Swaef et al. 2015). To date, however, little is known about stem radial variations and intra-annual growth dynamics of saplings in comparison to mature trees of the same species co-occurring within the same stand, although there is evidence that functional processes change with tree size (Mencuccini et al. 2005), affecting the timing, duration, and rate of intra-annual radial growth (Rossi et al. 2008; Rathgeber et al. 2011). Small trees also have minor ability to buffer their interactions with the environment due to lower biomass for storage (Lachenbruch et al. 2011; Scholz et al. 2011).

Consequently, the main focus of this study was to evaluate the influence of tree size (i.e., saplings vs. mature trees) on temporal radial growth dynamics and water status-related stem radius variations. A dry-mesic and a xeric sites were selected where saplings and mature trees of the same coniferous species co-occur. Norway spruce (*Picea abies* (L.) Karst.) and Scots pine (*Pinus sylvestris* L.) were selected at the dry-mesic and xeric sites, respectively. These coniferous species show different successional and phenological traits. *P. abies* is a late successional, moderately shade-tolerant and drought sensitive species, while *P. sylvestris* is a drought tolerant, light-demanding species dominating in the early successional stages (Ellenberg and Leuschner 2010; Lévesque et al. 2013). The study was conducted during two consecutive years, including the year 2015, which was Earth’s warmest year, since modern record-keeping began in 1880 (http://climate.nasa.gov/news/2391/; Jan 20, 2016). Because several authors (Cuny et al. 2012; Oberhuber et al. 2014) reported that intra-annual radial growth peaked synchronously in co-occurring mature coniferous species, I expected that (i) radial growth of co-occurring saplings and mature trees of both species culminates at the same time, and (ii) saplings exhibit higher SSRV and higher sensitivity of SSRV to environmental drivers than mature trees due to lower competition for water and earlier exhaustion of stem water reserves.

## Materials and methods

### Study plots

The study area is part of a postglacial rock-slide area situated in the montane belt (*c.* 750 m asl) within the inner Alpine dry valley of the Inn River (Tyrol, Austria, 47°13′53″ N, 10°50′51″ E). The human impact in this area was generally restricted to sporadic gathering of firewood and livestock grazing. The mean annual air temperature and total precipitation amount to 7.3 °C and 716 mm, respectively (long-term mean during 1911–2010 at Ötz, 812 m asl, 5 km from the study area). Shallow soils of the protorendzina type, i.e., rendzic and lithic leptosols, according to the FAO classification system (FAO 2006), are developed and consist of unconsolidated, coarse-textured materials with low water holding capacity. Two stands were selected, a south-facing xeric plot on a steep slope and a dry-mesic plot in a hollow facing north (Table 1). Study plots were situated <100 m (in linear distance) from each other. The dominating plant community at the xeric site is an open Spring Heath-Pine wood, whereas on the dry-mesic site, a mixed stand composed of *P. sylvestris* (60 %), *P. abies* (20 %), and *Larix decidua* (20 %) is developed. On the south-facing xeric site, pioneer vegetation prevails in the ground flora, whereas crowberry (*Vaccinium vitis*–*idaea* L.) and a thick moss layer dominate the understory at the dry-mesic plot, indicating slightly moist conditions at the latter site. The stem diameters of selected saplings and mature trees were *c.* 2.3 cm and *c.* 24 cm, respectively, and were not significantly different among species (Table 1). Although the stem height did not differ significantly among saplings, mature trees were twice as tall at the dry-mesic compared with the xeric site, which also indicates more favorable soil moisture conditions at the former site (Table 1). The mean tree age of selected mature trees and saplings was estimated to be >100 and <20 years, respectively (cf. Oberhuber and Gruber 2010; Schuster and Oberhuber 2013a, b). The canopy coverage of selected stands was 30 % at the xeric site and 70 % at the dry-mesic site. Light-demanding *P. sylvestris* and moderately shade-tolerant *P. abies* rejuvenate naturally under the canopy layer of the xeric and dry-mesic sites, respectively.

**Table 1:** Topographic and structural features of the study sites (<100 m in linear distance) and characteristics of *Pinus sylvestris* and *Picea abies* trees selected for dendrometer measurements

Species	Aspect	Slope (°)	CC (%)	Soil depth (cm)	Stem height^1^ (m)		SDM^1,2^ (cm)		Bark width (mm)	
					Saplings	Mature trees	Saplings	Mature trees	Saplings	Mature trees
*Picea abies*	N	<5	70	15–20	0.99 ± 0.42^a^	12.7 ± 1.6^a^	2.0 ± 0.8^a^	24.8 ± 4.8^a^	1.8 ± 0.4^a^	3.6 ± 0.5^a^
*Pinus sylvestris*	SSO	25–30	30	5–10	1.30 ± 0.29^a^	5.7 ± 0.9^b^	2.6 ± 0.6^a^	23.7 ± 3.1^a^	1.6 ± 0.2^a^	1.6 ± 0.4^b^

Mean values ± standard deviation are presented

*CC* canopy coverage, *SDM* stem diameter

Statistically significant differences of the mean values between species (independent samples) are indicated by different letters (*P* < 0.01; Student’s *t* test)

^1^Stem height and SDM measured before onset of growing season in March 2014

^2^SDM of saplings and mature trees measured at 10 cm above ground and breast height, respectively

### Recording stem radius variation

In autumn 2013, we installed temperature-compensated electronic band and diameter dendrometers with resolutions of <3 µm (DC2, DD-S, Ecomatik, Munich, Germany) on mature trees at breast height (*c.* 1.3 m) and on saplings at *c.* 15 cm stem height (*n* = 6–8 per species and size class). The temperature coefficient of the sensor was <0.1 µm/K. The measuring cable of the band dendrometers consisted of Invar-steel, with a temperature coefficient of linear expansion <1 µm/mK. Individual trees were randomly selected, but trees with major stem or crown anomalies were excluded. The dead outermost layers of the bark (periderm) were slightly removed to reduce the influence of hygroscopic swelling and shrinkage of the bark on the dendrometer records (DMRs) and to ensure close contact with the stem. Data loggers were programmed to record measurements every 30 min and daily SRV were calculated by averaging all daily measurements (‘daily mean approach’, Deslauriers et al. 2007) to represent a combination of water- and growth-induced radius changes (e.g., Herzog et al. 1995; Steppe et al. 2005). To separate daily patterns of water movement from irreversible expansion growth, the Gompertz function was applied to describe the long-term development of the radial increment over the entire growing season (e.g., Rossi et al. 2003). By applying this method, it is assumed that radial growth occurred constantly throughout the growing season, i.e., including periods of stem radius contraction, which is supported by the finding that growth onset within the study area occurs even when non-fully hydrated conditions prevail (Swidrak et al. 2011) and by the weekly monitoring of wood formation within the study area (Gruber et al. 2010; Swidrak et al. 2014). Standardized stem radial variations (SSRVs) were calculated by removing the long-term growth trend from the data, i.e., the Gompertz-modeled daily growth was subtracted from SRV. SSRV represents reversible shrinkage and swelling of tissues outside the cambium, which is considered to contribute the most to the stem water storage capacity (De Swaef et al. 2015). Positive SSRV values indicate water replenishment of the expandable tissues outside the cambium when tree water status is high (stem swelling), and negative SSRV values indicate that water stored in expandable tissues outside the cambium is translocated to the xylem (stem shrinkage). Although temperature variations may also contribute to SRV, several authors reported that these variations are insignificant (e.g., Zweifel et al. 2001; Steppe et al. 2005). Radial stem increments were defined as that part of the stem’s circadian cycle when the stem radius exceeded the morning maximum until the subsequent maximum (Deslauriers et al. 2003). When several days were required until the previous cycle maximum was exceeded, which was occasionally caused by drought-induced reversible shrinking of the stem, the radial increment was assumed to have occurred on the last day. This approach is justified considering the small amount of daily radial growth occurring within the study area. The non-linear regression procedure included in the Origin software package (OriginLab Corporation, Northampton, MA, USA) was applied to calculate the Gompertz function. Increment cores and stem discs of mature trees and saplings, respectively, were collected during a cool moist period in the fall to determine the bark width, excluding the non-functional periderm.

### Microclimate records

During the study period, air temperature, relative air humidity (RH), and daily precipitation were collected automatically (ONSET, Pocasset, MA, USA) at a 2-m height on an open ridge, i.e., in a non-vegetated area of the xeric plot. This site was chosen to record total rainfall by minimizing interception loss. In addition, air temperature and RH were recorded below the canopy within the dry-mesic stand at a 2-m height. The measuring intervals for all sensors were 30 min and the mean daily air temperatures were calculated by averaging all measurements (48 values per day). The vapor pressure deficit of the air (VPD) was calculated from the half-hourly records of air temperature and RH using the equation presented in Prenger and Ling (2000). The soil water content (SWC) in the 5- to 10-cm soil depth layer was recorded in both stands (ThetaProbes Type ML2x, Delta-T, Cambridge, England). The measuring intervals were set to 60 min, and the mean daily SWC (Vol.%) was calculated by averaging all measurements from three sensors per plot.

### Environmental influence on stem daily variation

Time series of the environmental variables (precipitation, RH, VPD, air temperature, and SWC) were compared with daily time series of SSRV using Pearson product-moment correlation statistics for the common period April–September. Kolmogorov–Smirnov tests were used to check that the selected variables were normally distributed. The Kendall rank correlation coefficient (*τ*) was determined for non-normally distributed variables. The software package used for the analyses was SPSS, version 21.0.0.1 (IBM SPSS Statistics, USA).

### Environmental variables during the 2014 and 2015 growing seasons

**Fig. 1:**
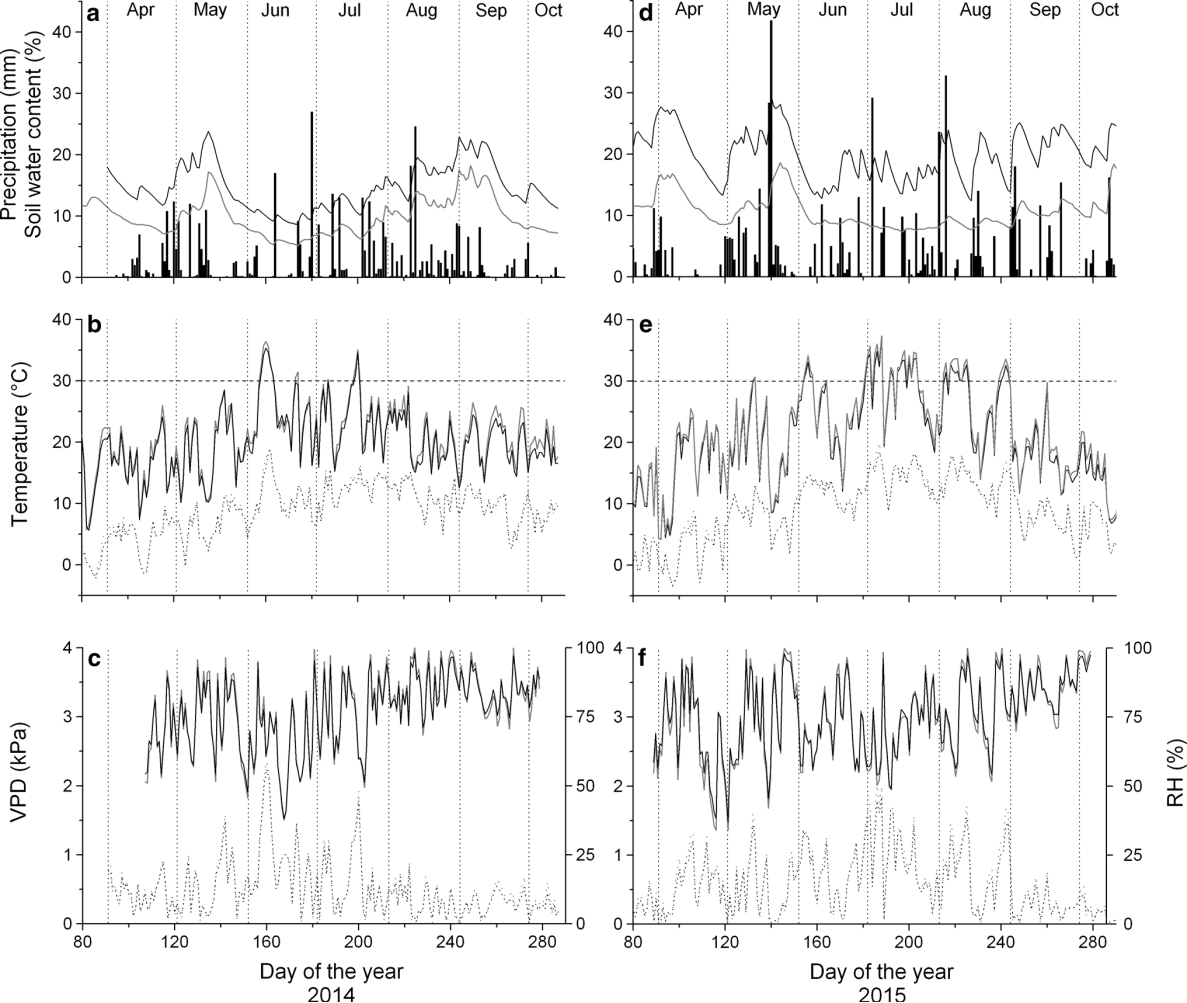
Climate variables and soil water content (vol.%) recorded during the 2014 (**a**–**c**) and 2015 (**d**–**f**) growing seasons within the study area. **a**, **d** Daily precipitation sum (*bars*) and soil water content (*lines*). **b**, **e** Maximum (*solid line*) and minimum (*dotted line*) daily air temperatures. An air temperature of 30 °C is indicated by the *horizontal dashed line*. **c**, **f** Relative air humidity (RH; *solid line*) and vapor pressure deficit (VPD; *dotted line*). *Black* and *grey lines* in **a**–**f** indicate environmental parameters recorded below canopy at the dry-mesic site and in an open non-vegetated area at the xeric site, respectively

At the start of the growing season in April, warm temperatures prevailed in both study years, which were 2.6 °C (2014) and 1.6 °C (2015) higher than long-term mean values (LTM; Fig. 1; Table 2). In 2014, the May and summer temperatures largely corresponded to the LTM. In 2015, however, exceptionally high temperatures were recorded during June through August. The mean daily air temperature during this period was 3.2 °C warmer than the LTM. The daily maximum air temperatures exceeded 30 °C on 41 days in 2015 compared with 11 days in 2014. The highest daily maximum air temperature reached 36.4 °C in 2014 (day of the year [doy] 160) and 37.4 °C in 2015 (doy 188). Precipitation was *c.* 30 % higher than the LTM in April 2014 and notably similar to the LTM in 2015 (Table 2). Only sporadic rainfall events occurred in April 2015, which caused a decrease in the SWC in the 5- to 10-cm soil depth layer at the end of April, i.e., to 13 and 9 vol.% at the dry-mesic and xeric sites, respectively (Fig. 1). Summer (June–August) precipitation was *c.* 20 % lower than the LTM in both study years. During April through September, the mean SWC in the 5- to 10-cm soil depth was 17 vol.% at the dry-mesic site and 10 vol.% at the xeric site (Fig. 1). High evapotranspiration demand during the exceptionally hot summer of 2015 most likely caused a largely constant SWC to prevail at the steep south-facing xeric site, which is prone to surface runoff during thundershowers. Within the study area, the mean VPD/RH during April–September was 0.52 kPa/76.2 % in 2014 and 0.65 kPa/73.8 % in 2015. VPD values >1.5 kPa in 2014/2015 were recorded on 6/12 days at the xeric site and on 5/8 days at the dry-mesic site (Fig. 1).

**Table 2:** April, May, and summer (June–August) mean daily air temperature and precipitation sum recorded within the study area

	Air temperature (°C)			Precipitation (mm)		
	1981–2010	2014	2015	1981–2010	2014	2015
April	7.8 ± 1.9	10.4 ± 2.9	9.4 ± 4.7	38 ± 21	52	34
May	12.7 ± 1.6	12.5 ± 4.1	13.4 ± 3.9	67 ± 31	63	155
Summer	16.5 ± 0.9	17.0 ± 3.3	19.7 ± 3.7	328 ± 65	267	268

Long-term values (1981–2010) were available from a nearby meteorological station (Oetz, 5 km from the study area). Mean values ± standard deviations are shown

## Results

**Fig. 2:**
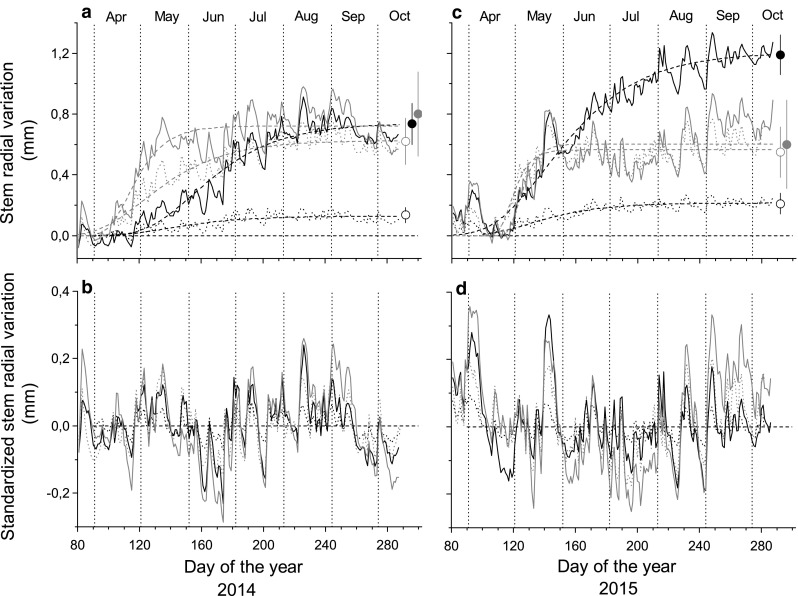
Time series of mean daily dendrometer records modeled by applying the Gompertz function (**a**, **c**), and standardized stem radial variations (**b**, **d**). Species are denoted by *black* (*P. abies*) and *grey* (*P. sylvestris*) *lines*, and mature trees and saplings are indicated by *solid* and *dotted lines*, respectively. The mean standard deviation between dendrometer records in **a** and **c** is indicated by *symbols*, whereby *black* and *grey closed circles* indicate mature *P. abies* and *P. sylvestris*, respectively, and *black* and *grey open circles* indicate *P. abies* and *P. sylvestris* saplings, respectively

**Fig. 3:**
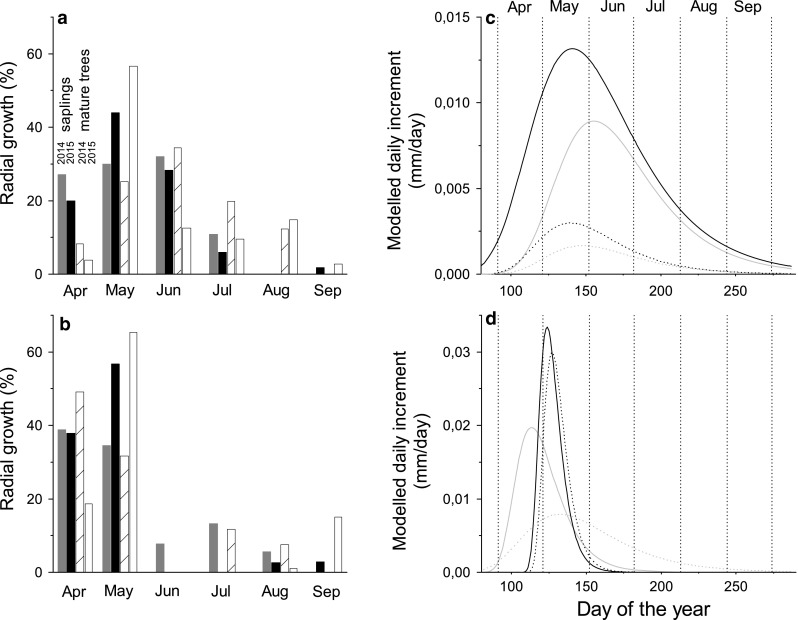
Radial growth of saplings and mature trees of *P. abies* (**a**, **c**) and *P. sylvestris* (**b**, **d**) during the 2014 and 2015 growing seasons, calculated from stem radial variations (**a**, **b**) and the Gompertz-modeled growth function (**c**, **d**; for details, see “Materials and methods” and Fig. 2 a, c). Study years in **c**–**d** are denoted by *grey* (2014) and *black lines* (2015), and mature trees and saplings are indicated by *solid* and *dotted lines*, respectively

Stem daily variations in 2014 and 2015 are compared among the *P. sylvestris* and *P. abies* saplings and mature trees in Fig. 2a and c. Strikingly depressed radial growth of saplings compared with mature trees is quite obvious for *P. abies* in both study years. Contrary to this, the radial growth of *P. sylvestris* was notably similar among the mature trees and saplings (Fig. 2a, c). Irrespective of tree size, radial stem growth significantly increased (*P* < 0.05; Student’s *t* test) by *c.* 65 % in *P. abies* in 2015 compared to 2014. Radial stem growth of *P. sylvestris* (saplings and mature trees) slightly decreased by *c.* 10 and 15 % in the saplings and mature trees, respectively (decline not statistically significant). *P. abies* and *P. sylvestris* differed distinctly in the temporal dynamics of radial growth (Figs. 2, 3). Based on the calculated Gompertz functions, the maximum daily radial growth of the saplings and mature *P. sylvestris* trees peaked between the end of April and early May (doy 113–123), i.e., within a rather short time period. Radial growth of the *P. abies* saplings culminated in mid-May compared with the end of May/early June for the mature trees (Table 3). Hence, there was a difference in the timing of maximum radial growth of almost 2 weeks between the saplings and mature trees of *P. abies*. Decreasing SWC in late May through early June (Fig. 1) coincided with the abrupt decrease in the radial growth of the mature *P. sylvestris* trees (Fig. 2). In the saplings and mature *P. abies*, the main growing season extended from April through June and May–June, respectively (Fig. 3a). Due to the onset of abrupt growth in mature *P. abies* in late April 2015, the Gompertz-modeled daily increment overestimated growth at this time (Fig. 2c). In *P. sylvestris*, the main growing period was concentrated in April–May irrespective of tree size (Fig. 3b, d).

**Table 3:** Parameters of the Gompertz function for 2014 and 2015 (see Fig. 2) intra-annual radial growth of *Pinus sylvestris* and *Picea abies*, and *R* ^2^ of the model

Species	Year	*A* (µm)	*I*_p_ (doy)	*κ*	*R* ^2^
*Pinus sylvestris*					
Saplings	2014	626 ± 10	123 ± 1.4	0.034 ± 0.002	0.896
	2015	565 ± 8	118 ± 0.9	0.144 ± 0.022	0.818
Mature trees	2014	721 ± 10	113 ± 1.2	0.074 ± 0.009	0.836
	2015	603 ± 12	123 ± 1.3	0.151 ± 0.035	0.649
*Picea abies*					
Saplings	2014	128 ± 5	138 ± 3.0	0.035 ± 0.005	0.661
	2015	215 ± 4	130 ± 1.6	0.038 ± 0.003	0.828
Mature trees	2014	739 ± 14	155 ± 1.4	0.033 ± 0.002	0.935
	2015	1206 ± 16	141 ± 1.1	0.030 ± 0.002	0.933

Mean values ± standard deviation

*A* upper asymptote, *I*_*p*_ inflection point, *doy* day of the year, *κ* rate of change parameter

**Fig. 4:**
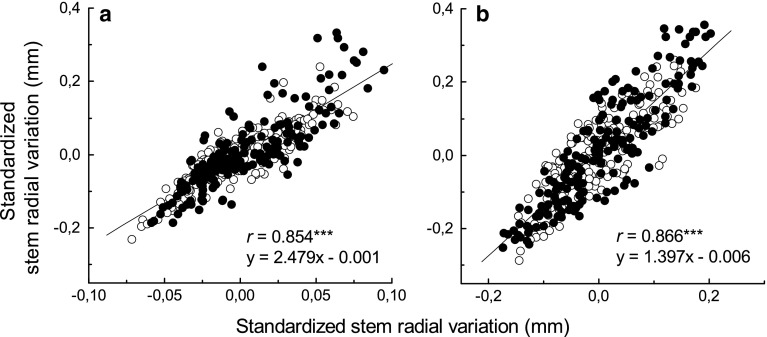
Relationships between standardized stem radial variation (mm) of **a** mature *P. abies* vs. saplings and **b** mature *P. sylvestris* vs. saplings. *Open* and *filled circles* indicate the 2014 and 2015 data, respectively. The Pearson correlation coefficients *r* and regression equations are indicated (***P < 0.001). Both study years were included when calculating the linear functions

Rather homogenous fluctuations of SSRV among species and tree size are obvious in Fig. 2b and d, which correspond to close to linear relationships observed between the SSRV of mature trees vs. saplings in both species (Fig. 4). However, the change in SSRV (i.e., slope of the relationship) was 2.48- and 1.40-fold higher for the mature *P. abies* and *P. sylvestris* trees compared with their respective saplings (Fig. 4a, b), indicating more intense exploitation of water stored outside the cambium in mature trees, especially *P. abies*. The higher SSRV of the mature trees compared with the saplings was also indicated by higher frequencies and magnitudes of negative SSRV values in the former (Online Resource 1).

**Fig. 5:**
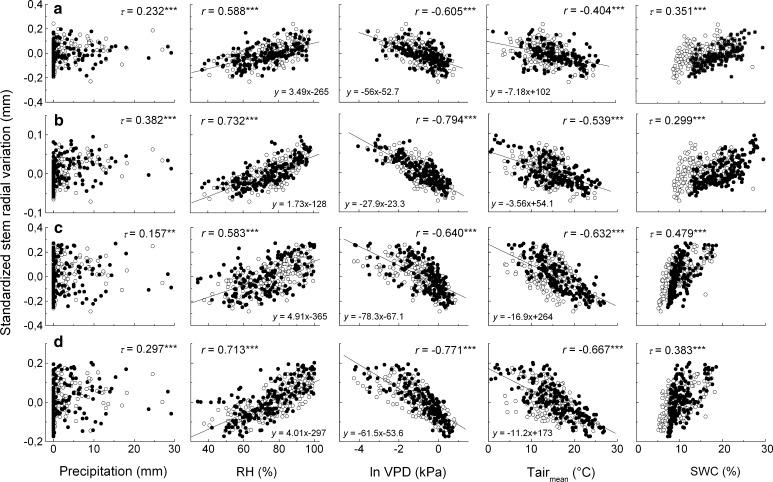
Correlations between standardized stem radial variation of *P. abies* (**a** mature trees, **b** saplings) and *P. sylvestris* (**c** mature trees, **d** saplings) and environmental variables from April through September 2014 (*open circles*) and 2015 (*closed circles*). For clarity, regression equations are presented in micrometer. A 1-day lag in SSRV was considered in the correlation with SWC (*RH* relative air humidity, *VPD* vapor pressure deficit, *Tair*_*mean*_ mean daily air temperature, *SWC* soil water content). The Pearson correlation coefficient (*r*) and Kendall rank correlation coefficient (*τ*) were calculated by including data of both years (*n* = 366; **P < 0.01, ***P < 0.001)

As depicted in Fig. 5a–d, the SSRV of both species and size classes showed significant responses to environmental drivers. The closest direct correlations were found with RH, with mean Pearson correlation coefficients (*r*) of 0.722 and 0.585 for the saplings and mature trees, respectively (*P* < 0.001). Highly significant inverse relationships were also observed with VPD for both species, and the correlation coefficients were distinctly higher for the saplings (mean *r* = −0.782) compared with the mature trees (mean *r* = −0.622). Higher coefficients were found between the mean air temperature and the SSRV of the *P. sylvestris* saplings and mature trees (mean *r* = −0.649) compared with *P. abies* (mean *r* = −0.471). Kendall correlation coefficients (*τ*) between SSRV and precipitation and SWC were statistically significant and ranged between 0.157 (*P* < 0.01) and 0.479 (*P* < 0.001). The slopes of the linear Pearson correlations were distinctly influenced by tree size in *P. abies* (Fig. 5a, b), while minor effects of tree size on the sensitivity of SSRV to environmental drivers were found in *P. sylvestris* (Fig. 5c, d).

## Discussion

### Temporal dynamics of radial growth

The early culmination of radial growth in mature *P. sylvestris* in late April/early May is consistent with the previous findings within the study area (Gruber et al. 2010). This indicates that the radial growth of *P. sylvestris* at xeric sites is restricted to a short period in spring when soil water availability is rather low compared with more favorable growing conditions, i.e., an increase in precipitation during summer. Gruber et al. (2010) suggested that xeric conditions might require an early switch of carbon allocation to belowground organs and mycorrhiza. Because the same temporal growth dynamics were found in the saplings and mature trees during both study years, tree size did not affect growth response of *P. sylvestris* to xeric conditions. Conversely, in *P. abies*, maximum radial growth peaked approximately 2 weeks earlier in the saplings compared with the mature trees, and the radial increment differed more than fivefold among the saplings and mature trees. Several authors found that water stress causes earlier culmination and ending of radial growth in conifers (e.g., Pichler and Oberhuber 2007; Levanič et al. 2009; Thabeet et al. 2009; Gruber et al. 2010), and Deslauriers et al. (2016) reported that water availability is the most important factor for wood formation to occur. Furthermore, Oberhuber et al. (2014) found that the intra-annual radial growth of co-occurring *P. abies* and *P. sylvestris* at a dry-mesic site peaked synchronously. Therefore, as I hypothesized, the earlier culmination of radial growth in *P. sylvestris* than in *P. abies* reported in this study can be related to the earlier development of a more constrained stem water status at the xeric site. In this way, we also interpret different timings in the culmination of radial growth among co-occurring saplings and mature *P. abies*; however, this is in contrast to expectations. My reasoning is consistent with the findings of Oberhuber et al. (2015) within the study area, i.e., that saplings exhibited a higher stem water deficit than mature *P. abies* individuals in response to limited water availability during the growing season. Although Muller et al. (2011) reported that tree growth under water stress is controlled by sink limitation (i.e., inhibition of cell division) rather than source limitation (i.e., reduction of photosynthesis), the impaired carbon status of moderately shade-tolerant *P. abies* might contribute to strikingly low radial growth in saplings. The involvement of impaired carbon status in saplings also seems, likely because *P. abies* is an isohydric species, i.e., an excessive decrease in leaf water potential by the early stomatal closure is prevented (McDowell et al. 2008), and source limited growth of seedlings and saplings was found in the forest understory (Körner 2003; Lloyd and Farquhar 2008).

Surprisingly, exceptionally high air temperatures prevailing in July through August 2015 did not adversely affect the radial growth of either species or size class, which can be explained by the early decline in daily growth rates in May and June in *P. sylvestris* and *P. abies*, respectively, and high precipitation in summer. A strong dependency of radial stem growth on precipitation during late spring and the preconditioning of tree vigor in previous years has repeatedly been reported for this study area (e.g., Pichler and Oberhuber 2007; Schuster and Oberhuber 2013b). Hence, exceptionally high air temperatures during summer do not limit the radial growth of trees, which exhibit maximum growth rates in spring. However, lag effects on tree vigor and growth have to be considered.

### Influence of tree size and environmental factors on reversible water translocation

Maximum daily growth rates of *c.* 30 µm and *c.* 15 µm were recorded for *P. sylvestris* and *P. abies*, respectively, whereas the water-related daily changes in growth-detrended stem radial variations (SSRV) amounted to >300 µm. This indicates that the calculated SSRV reflects changes in stem hydration rather than changes in actual radial growth. The finding that especially, mature *P. abies* trees exploited water stored outside the cambium to a greater extent than saplings is consistent with the results of a dendroecological study, which revealed that large *P. abies* trees showed highly significant responses to precipitation, while small trees were insensitive to precipitation in the current year (Schuster and Oberhuber 2013a). However, our results are in contrast to a previous study, where a higher stem water deficit was found in saplings compared with mature *P. abies* (Oberhuber et al. 2015). This discrepancy can be explained by the different ways that original SRV values were detrended for long-term growth to SSRV. In our previous study, we assumed that radial growth was restricted to short periods when the stem was fully hydrated, i.e., periods of incomplete stem radius recovery due to drought-induced stem shrinkage were ignored (cf. Ehrenberger et al. 2012). In this study, radial stem growth was expected to occur throughout the growing season according to an asymmetric sigmoid curve (Gompertz function), i.e., cambial cell division and enlargement were assumed to take place even when the stem was not fully hydrated. This reasoning is supported by findings that (i) growth resumption within the study area in spring is triggered by air temperature rather than precipitation and occurs even when the stem is not fully hydrated (Swidrak et al. 2011); (ii) temporal dynamics of wood formation (xylogenesis) in *P. abies* and *P. sylvestris* could be accurately modeled by applying the Gompertz function (*R* ^2^ > 0.980; Gruber et al. 2010; Swidrak et al. 2014); and (iii) the maximum SSRV reached at most 10 % of the bark width (excluding the periderm) of *P. abies* and 25 % of the bark width of *P. sylvestris*, indicating that water stored outside the cambium was not depleted even at the xeric site.

Because transpiration is coupled with xylem sap flow (e.g., Steppe et al. 2005) and because a close lateral linkage exists between xylem and phloem (e.g., Zweifel and Häsler 2001), changes in tree water status are directly converted to reversible SRV. Chan et al. (2016) reported that radial changes are approximately linearly proportional to changes in stem water potential. Hence, close correlations between atmospheric conditions and SSRV found for the saplings and mature trees of both species indicate that transpiration strongly draws upon water reserves from the elastic tissues of the bark, and the stem reservoirs are rapidly replenished when evaporative demand declines. This observation is in line with several other studies (e.g., Zweifel et al. 2005; Turcotte et al. 2011; Ehrenberger et al. 2012) and suggests that elastic tissue reservoirs contribute to the avoidance of the early stomatal regulation under increasing soil drought (Čermak et al. 2007). The statistically significant but lower correlation coefficients found between SSRV and SWC, as well as SSRV and precipitation corroborate, reports that transpiration primarily draws upon internal storage tissues in the stem rather than soil water to be able to respond rapidly to changes in atmospheric demand (e.g., Hinckley and Bruckerhoff 1975; Čermak et al. 2007; for a review, see Fernández and Cuevas 2010). However, it has to be considered that the SWC recorded at 5- to 10-cm soil depth did not encompass the complete rooting zone of the mature trees, which may have taken up water from the deep cracks within the rocky study area. The slopes of the linear regressions between the environmental variables and SSRV revealed that throughout the growing season (April-September), the SSRV of the mature trees was more strongly affected by environmental conditions (RH, VPD, and air temperature) compared with the SSRV of the saplings, especially in *P. abies*. This finding is consistent with the development of higher SSRV in mature trees compared with saplings and with dendroclimatological studies revealing higher climate sensitivity of older and larger *P. abies* trees (Mèrian and Lebourgeois 2011; Schuster and Oberhuber 2013a).

The finding that the small trees were less sensitive to water availability than the large trees and that they less intensively exploited the stem water reservoirs to maintain tree water status and transpiration compared with the mature trees was unexpected. The influence of higher radiation and evaporative demand experienced by exposed crowns was proposed to explain the more pronounced drought sensitivity of larger trees in forests worldwide (Bennett et al. 2015). Canopy density strongly differed among the study sites (canopy coverage was 70 and 30 % at the dry-mesic and xeric site, respectively), and stand heights were *c.* 16 m at the dry-mesic site and *c.* 5 m at the xeric site. These differences in canopy structure most likely explain the varying influences of tree sizes on SSRV among sites, because diverse (micro)climate conditions for saplings and mature trees are expected at the dry-mesic site, while quite similar conditions might prevail at the xeric site. Hence, the hypothesized higher sensitivity of saplings to climate due to lower competitiveness for water in addition to lower stem water reserves was most likely outweighed by more favorable atmospheric conditions, i.e., reduced evaporative demand below the canopy, especially at the dry-mesic site. Consistently, the notably similar slopes of the linear relationships between the atmospheric conditions and the SSRV of the saplings and mature *P. sylvestris* were related to the open canopy at the xeric site. This is corroborated by the results of von Arx et al. (2012, 2013), who reported that the moderating capacity on the microclimate is related to canopy density and site-specific water availability, and is lower in pine forests than in broadleaved and non-pine conifer forest ecosystems.

## Conclusions

This study provides new insights regarding the influence of tree size and site conditions on the temporal dynamics of radial growth and the use of stored water to maintain transpiration under conditions of high evaporative demand. The results revealed that (i) the development of a canopy atmosphere mitigates water stress experienced by saplings and (ii) high temperatures in summer do not considerably affect the current year growth of saplings and mature conifers, which exhibit an early culmination of radial growth in spring. These findings are important to accurately model size-dependent sensitivity of trees to drought.

### **Author contribution statement**

WO conceived the analysis, analyzed, and interpreted data and wrote the paper.

## Electronic supplementary material

Below is the link to the electronic supplementary material.

Supplementary material 1 (PDF 409 kb)
