# Supplementary material for: Soil water availability and evaporative demand affect seasonal growth dynamics and use of stored water in co-occurring saplings and mature conifers under drought
Source: Trees (Berl West). 2016 Sep 29;31(2):467–78. doi: 10.1007/s00468-016-1468-4 (PMC5375970; doi:10.1007/s00468-016-1468-4)
Supplement: Supplementary file 1 — Supplementary material 1 (PDF 409 kb) [file 468_2016_1468_MOESM1_ESM.pdf]

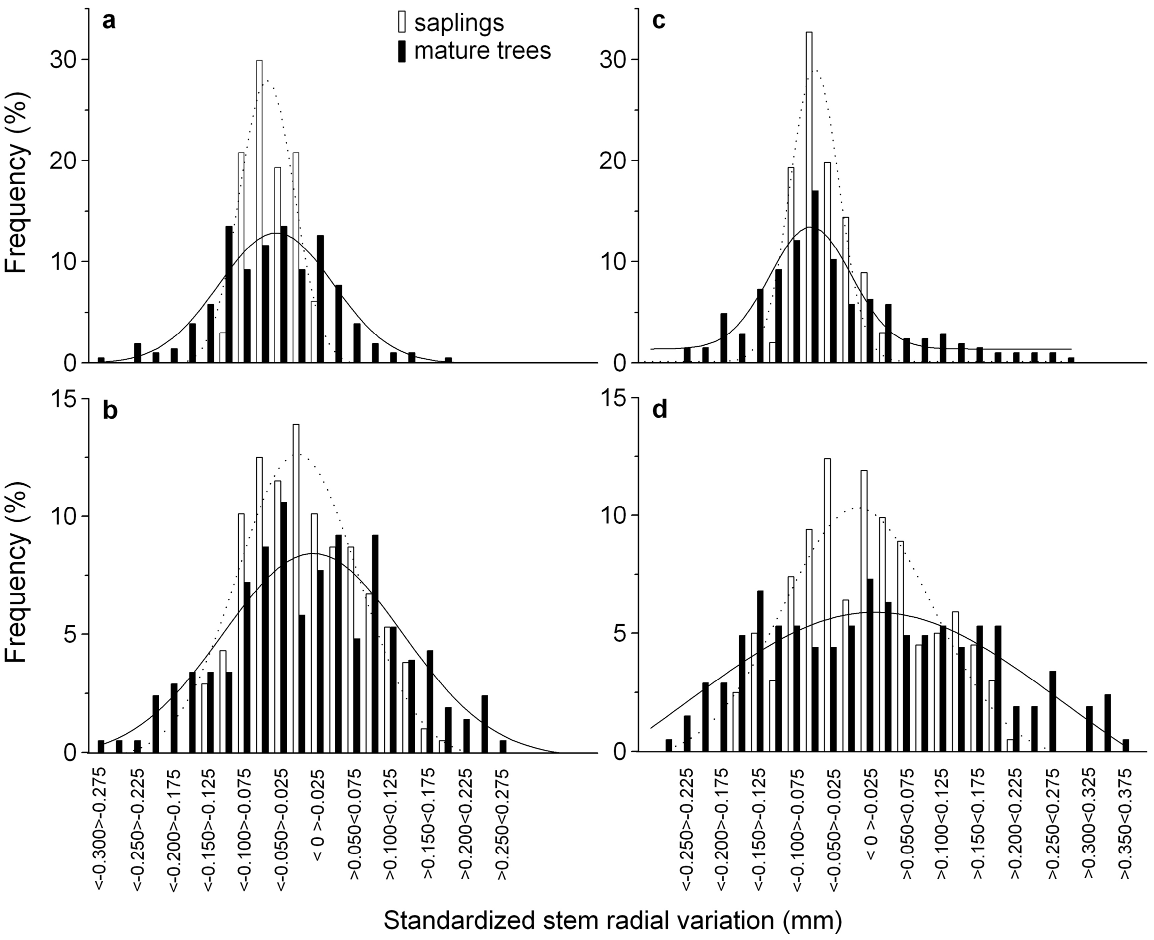


**Online Resource 1** Frequency distribution of standardized stem radial variation of saplings (open bars) and mature trees (closed bars) in 2014 (a, b) and 2015 (c, d) of *Picea abies* (a, c) and *Pinus sylvestris* (b, d). Solid and dotted lines indicate Gaussian fitting for mature trees and saplings, respectively. Note different scaling of the y-axes

**Soil water availability and evaporative demand affect seasonal growth dynamics and use of stored water in co-occurring saplings and mature conifers under drought**

Trees – Structure and Function

Walter Oberhuber

Institute of Botany, Leopold-Franzens-University of Innsbruck, Sternwartestrasse 15,

A-6020 Innsbruck, Austria

Walter.Oberhuber@uibk.ac.at
